# Study on the Complex Melanoma

**DOI:** 10.3390/cancers16050843

**Published:** 2024-02-20

**Authors:** Anna Fateeva, Suzie Chen

**Affiliations:** 1 Susan Lehman Cullman Laboratory for Cancer Research, Rutgers University, Piscataway, NJ 08854, USA; anna.fateeva@rutgers.edu; 2 Graduate Program in Cellular and Molecular Pharmacology, Rutgers University, Piscataway, NJ 08854, USA; 3 Rutgers Cancer Institute of New Jersey, New Brunswick, NJ 08901, USA

Melanoma only accounts for about 1% of cases in skin cancer, unlike basal cell and/or squamous cell carcinomas; however, it owes its notoriety to being the deadliest type of skin cancer [1]. Significant advancement in melanoma therapies in the past two decades have enabled the development of cutting-edge therapies, including novel targeted therapeutic agents and immunotherapies, such as immune checkpoint inhibitors and oncolytic viruses [2]. However, onset of resistance and lack of response to one or more of these latest treatments in many melanoma patients have motivated investigators to seek greater improvements in treatment modalities for better melanoma patient outcomes. Many such improvements are combinations of currently available options; however, despite some advantages, such as the enhanced efficacy offered by these novel options, they are often accompanied by increased toxicity. Thus, metastatic melanoma remains a difficult disease to manage, with a 5-year survival rate of about 35% [3]. This is mostly due to the complexity of the processes of neoplasm formation and progression, as well as tumors’ adaptability and capability to develop resistance to therapeutic interventions. For this Special Issue, entitled “Study on the Complex Melanoma”, fifteen articles were selected to showcase the complexity of melanoma and highlight the areas that still require a better understanding of the disease to design improved therapeutic strategies in the treatment approaches.

Due to this complexity, the ability to use prognostic markers to better predict outcomes and to adjust treatment options in a more personalized manner is of high significance. Kelly et al. introduced a Metastatic Uveal Melanoma Prognostic Risk Score (MUMPS) for patients with metastatic uveal melanoma (UM) who were treated with anti-PD1/L1 and/or anti-CTLA4 immunotherapy in 2014–2019. MUMPS was developed using a single-center retrospective cohort study to comprise three clinically available variables: time to metastatic diagnosis, the presence of bone metastases, and levels of lactate dehydrogenase in serum. They used multivariable Cox regression models and Kaplan–Meier log-rank tests to assess differences in progression-free survival and overall survival rates between treatment groups, identify these variables, and establish three types of prognoses: good, intermediate, and poor. However, the authors stressed that even though MUMPS has a good predictive potential to improve clinical decision-making, it requires further validation in independent datasets.

Olliver et al. reported on the incidence of the abscopal response (AR)—a patient response to radiation therapy outside of the irradiated site—and its therapeutic effects in patients with metastatic melanoma. They considered data from the patients who required radiation and exhibited disease progression on treatment with immune checkpoint inhibitors or without ongoing systemic treatment in the period from 1998 to 2020. The patients that had an observed AR were reported to have significantly longer progression-free survival and overall survival than patients without AR. They also observed that AR was more likely to develop in patients treated with immunotherapy. Even though AR is a rare event that occurred only in approximately 13% of patients in this sample group, the authors considered that it is still valuable to use AR as a prognostic marker to predict the outcome.

Focusing on the tumor microenvironment, Gutiérrez-Seijo et al. reported on profiles of tumor-associated macrophages (TAMs) in patients with metastatic cutaneous melanoma (CM). They assessed parameters such as density, location, size, and polarization marker expression of TAMs using confocal microscopy, although no correlation with the propensity for metastasis in CM was observed. However, when they measured the intracellular cytokine levels of CCL20, TNF, and VEGFA using single-cell multiparametric confocal microscopy, they found that high levels of these cytokines were strongly associated with metastasis and a worse prognosis in primary melanoma patients. The Kaplan–Meier method was used to analyze correlation with disease-free survival and overall survival. Moreover, the authors discovered that p53 and NF-kB pathways were involved in the regulation of these cytokines in TAM. They suggested that the specific TAM profiles could be used as prognostic markers for poor outcomes in CM patients, and that the addition of compounds targeting p53/NF-kB could be beneficial in patients with such TAM profiles.

In addition to improving medication precision and targeting it to the appropriate group of patients, it remains critical to identify novel approaches in melanoma treatment, as tumors constantly evolve and find new ways to change the status quo in the host. Huang et al. reported a promising new therapeutic agent for the treatment of CM. They showed that YK-4-279, an inhibitor of RNA helicase A, attenuates the progression of melanoma when administered both at the time and after tumor initiation. When given after tumor initiation, 29–60% of patients showed partial blockade or a delay in melanoma progression. However, the authors noted that given the lack of complete response, combining YK-4-279 with another therapeutic agent may increase the efficacy of the single agent YK-4-279 with synergistic outcomes.

Podgorska et al. reported the consequences of vitamin D hydroxy derivatives in melanoma cells. They showed that upon binding to vitamin D receptor (VDR), the activated forms of vitamin D, 1,25(OH)_2_D_3_, 20(OH)D_3_, and 1,20(OH)_2_D_3_, inhibited cell proliferation, migration, and the ability of cells to form colonies and spheroids in vitro. Following the knockout of VDR, increased but incomplete resistance to the action of vitamin D hydroxy derivatives was observed, suggesting the involvement of VDR and the activated forms of vitamin D in the suppression of melanoma cell growth. Additional work is needed to unravel the mechanisms of action, as are in vivo validation studies.

Eddy et al. described the unique role of mGluR1, metabotropic glutamate receptor 1, in driving melanoma development and progression. The authors summarized how mGluR1 was discovered; its role in the onset and progression of melanoma in vitro and in vivo; and the promotion of cell proliferation and cell survival by the activated receptor through downstream signaling pathways and effectors. Since the natural ligand, glutamate, for functional mGluR1 receptors is required to maintain the transformed phenotypes in vitro and in vivo, inhibitors that modulate glutamate levels were investigated. The roles of other metabotropic receptors in melanoma and the involvement of glutamatergic signaling in other cancers were also described.

Jansen et al. reported on the prevalence and impact on patient life quality of a complication after an existing surgical procedure—postoperative lymphedema following inguinal lymphadenectomy—in stage III melanoma patients. Inguinal lymphadenectomy is a surgical procedure in which lymph nodes are removed from the groin; postoperative lymphedema is a possible complication after the surgery that constitutes a buildup of fluid in the body. In this paper, the authors specifically considered surgeries conducted using a videoscoping approach with a few smaller incisions rather than an open approach when one large inguinal incision is made. After evaluating patients from 2015 to 2019, lymphedema incidence was found to be 40% at 3 months and increased to 50% at 12 months after the surgery. Quality of life was measured using the Lymph-ICF-LL questionnaire, which showed an initial increase in score indicating a decrease in life quality with subsequent further reductions at the 1-year mark. Notably, the score was higher for female patients and patients who received adjuvant radiotherapy.

With the increasing knowledge of skin cancer, more components have been identified that may contribute to the development of melanoma. Yamauchi et al. claimed that the significant increase in melanoma cases in recent years cannot be explained merely by increased UV exposure. They are advocating to examine social–environmental factors, such as alcohol consumption, to better understand melanoma epidemiology, and discuss molecular pathways underlying the role of ethanol in the development of cancers such as melanoma. They concluded that further research is required to improve the comprehension of other components in melanoma development and progression.

Chalada et al. examined the epidemiology of UM in two states in Australia—Queensland (QLD) and Victoria (VIC). The objective was to determine the age-standardized incidence rates (ASRs) of UM. Using cancer registry data from 2001 to 2013, they calculated world-standardized UM ASRs and incidence rate ratios (IRRs). They established that QLD had a 21% higher incidence of UM than VIC. The authors associated the difference with varying ultraviolent radiation susceptibility, indigenous populations, social behaviors, chemical exposure, and socioeconomic status. They emphasized the need for better sun-protective behaviors in regions with a higher chance of developing UM, especially in QLD, which is already known to have the highest ASRs for CM in the world.

Morales et al. emphasized the importance of considering the influences of the tumor microenvironment in treatment efficacy. They underlined the distinct consequences of cancer-associated fibroblasts and healthy fibroblasts in the therapeutic response to vemurafenib and cobimetinib in their studies. Cancer-associated fibroblasts attenuated the efficacy of the drugs in both 2D and 3D cell cultures, while healthy fibroblasts increased the drug efficiency.

With these advances in the field of melanoma therapy, it remains important to continue to expand our knowledge in the basic biology of melanocytes and melanoma cells to conceive improved therapeutic approaches based on rational strategies to deliver more effective treatment options and achieve better results in patients. Kline et al. reported on the ambiguous functions of selenoproteins in protecting melanocytes from reactive oxygen species (ROS), thus delaying the onset and progression of melanoma. Previously, it was found that an increase in selenoprotein activity led to protection against oxidative damage; however, later it was discovered that a selenoprotein thyioredoxin reductase 1 (TR1) is associated with melanoma progression and metastasis. Here, the authors reported that the depletion of TR1 resulted in the loss of melanocyte-specific master transcriptional factor, MITF, mediated via the modification of the redox state of the protein. This, in turn, led to the regulation of pigmentation and tumorigenesis, possibly through the reduced activities of tyrosinase, tyrosinase-like protein-1 expression, and other MITF-regulated genes.

Carpenter et al. reviewed antioxidant systems in cells, specifically melanocytes. They discussed the role of NRF2, nuclear factor erythroid 2-related factor, and its downstream targets for maintaining redox balance, as well as how cancer cells can co-opt these systems to their advantage to escape destruction by increasing their antioxidant capacity. The authors also speculated on the possible ways to utilize this co-option for therapeutic interventions.

Scheau et al. explored the roles of multiple endocrine factors, catecholamines, glutamate, serotonin, and cannabinoids, as well as multiple neurohormones, neuropeptides, mast cells, and nitric oxide in melanoma development and progression.

Neuffer et al. described the advantages of zebrafish as a model organism for studying pigment disorders, such as albinism and melanoma. They suggested that due to the high fecundity, visible melanin development in melanophores (melanocytes in mammals), and conserved melanogenesis pathways, zebrafish represent an economically favorable model organism to use for studying skin biology and disorders. Such a model could be an interesting alternative to commonly accepted rodent-based studies in the field.

Finally, melanoma is a disease that affects not only skin, but also mucous membranes; mucosal melanoma (MM) is a rare type of melanoma. Cazzato et al. described an even rarer subtype of mucosal melanoma—urological melanoma. They highlighted the need for the early diagnosis and identification of genetic alterations in the rare melanoma types. They also proposed that targeted therapy might be a good treatment option for some patients and the use of next-generation sequencing to identify specific driver mutations.

Overall, the collection of articles in this Special Issue discusses various aspects of melanoma biology and therapy: the development of prognostic markers for better personalized approaches for melanoma patients; the development of new melanoma therapeutic options; and epidemiology and risk factors that contribute to melanoma development. Additional contributors that should be considered include the tumor microenvironment, unexplored functions of proteins in melanoma cells, and rare types of melanomas, where better identification and treatment options are needed. Advancing the knowledge of the complexity of melanoma and better understanding of melanocyte and melanoma biology will provide rational approaches to design improved strategies for the treatment of this deadly disease.
